# *catena*-Poly[[bis­(dimethyl­formamide-κ*O*)cadmium]-bis­(μ-4-nitro­phenyl­cyanamido-κ^2^ *N* ^1^:*N* ^3^)]

**DOI:** 10.1107/S1600536812001924

**Published:** 2012-02-04

**Authors:** Hossein Chiniforoshan, Mehdi Jazestani, Behrouz Notash

**Affiliations:** a Department of Chemistry, Isfahan University of Technology, Isfahan 84456-38111, Iran; b Department of Chemistry, Shahid Beheshti University, G. C., Evin, Tehran 1983963113, Iran

## Abstract

In the title coordination polymer, [Cd(C_7_H_4_N_3_O_2_)_2_(C_3_H_7_NO)_2_]_*n*_, the Cd^II^ atom, lying on an inversion center, is six-coordinated in a distorted N_4_O_2_ octa­hedral geometry. The N atoms of the 4-nitrophenylcyanamide anions form the equatorial plane and the O atoms of the dimethyl­formamide mol­ecules occupy the axial positions. The anions act as bridging ligands, connecting the Cd atoms into a one-dimensional coordination polymer along [100].

## Related literature

For background to phenyl­cyanamide ligands and their complexes, see: Crutchley (2001 ▶). For polynuclear complexes of phenyl­cyanamide ligands, see: Ainscough *et al.* (1991 ▶); Chiniforoshan *et al.* (2009 ▶, 2010 ▶); Escuer *et al.* (2004 ▶). For the preparation of 4-nitro-phenyl­cyanamide used in the synthesis of the title compound, see: Crutchley & Naklicki (1989 ▶).
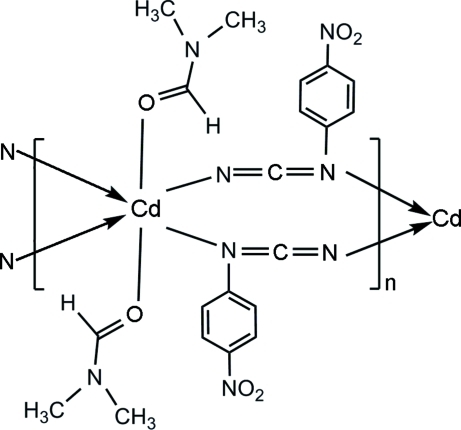


## Experimental

### 

#### Crystal data

[Cd(C_7_H_4_N_3_O_2_)_2_(C_3_H_7_NO)_2_]
*M* *_r_* = 582.87
Triclinic, 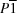

*a* = 5.6070 (11) Å
*b* = 9.811 (2) Å
*c* = 11.679 (2) Å
α = 67.44 (3)°
β = 81.93 (3)°
γ = 84.28 (3)°
*V* = 586.7 (2) Å^3^
*Z* = 1
Mo *K*α radiation
μ = 0.98 mm^−1^
*T* = 298 K
0.45 × 0.10 × 0.08 mm

#### Data collection

Stoe IPDS 2T diffractometer
Absorption correction: numerical (*X-SHAPE* and *X-RED32*; Stoe & Cie, 2002 ▶) *T*_min_ = 0.887, *T*_max_ = 0.923
6589 measured reflections
3150 independent reflections
3038 reflections with *I* > 2σ(*I*)
*R*_int_ = 0.066

#### Refinement

*R*[*F* ^2^ > 2σ(*F* ^2^)] = 0.044
*wR*(*F* ^2^) = 0.114
*S* = 1.11
3150 reflections
162 parameters
H-atom parameters constrained
Δρ_max_ = 0.82 e Å^−3^
Δρ_min_ = −0.89 e Å^−3^

### 

Data collection: *X-AREA* (Stoe & Cie, 2002 ▶); cell refinement: *X-AREA*; data reduction: *X-AREA*; program(s) used to solve structure: *SHELXS97* (Sheldrick, 2008 ▶); program(s) used to refine structure: *SHELXL97* (Sheldrick, 2008 ▶); molecular graphics: *ORTEP-3* (Farrugia, 1997 ▶); software used to prepare material for publication: *WinGX* (Farrugia, 1999 ▶).

## Supplementary Material

Crystal structure: contains datablock(s) I, global. DOI: 10.1107/S1600536812001924/hy2496sup1.cif

Structure factors: contains datablock(s) I. DOI: 10.1107/S1600536812001924/hy2496Isup2.hkl

Additional supplementary materials:  crystallographic information; 3D view; checkCIF report

## Figures and Tables

**Table 1:** Selected bond lengths (Å)

Cd1—N1^i^	2.287 (3)
Cd1—O3	2.347 (3)
Cd1—N2	2.383 (3)

Symmetry code: (i) 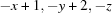.

**Fig. 1.**
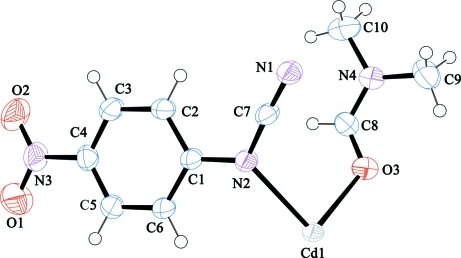
The asymmetric unit of the title compound with displacement ellipsoids drawn at 50% probability level.

**Fig. 2.**
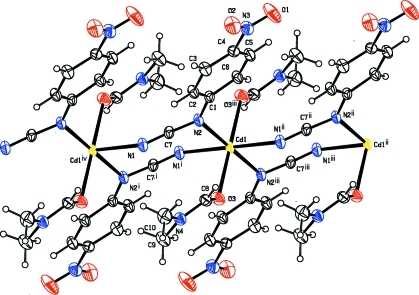
The one-dimensional polymeric structure of the title compound. [Symmetry codes: (i) 1-x, 2-y, -z; (ii) -1+x, y, z; (iii) -x, 2-y, -z; (iv) 1+x, y, z.]

## supplementary crystallographic information

### Comment

Phenylcyanmide ligands can act as monodentate, bidentate and also as bridging ligands (Crutchley, 2001). In the bridging mode, the cyanamido group (NCN) is coordinated in an end-to-end mode, forming polynuclear complexes (Ainscough *et al.*, 1991; Chiniforoshan *et al.*, 2009, 2010; Escuer *et al.*, 2004).

Following our work with this family of ligands, we report here the synthesis and crystal structure of a cadmium(II) coordination polymer of 4-nitro-phenylcyanamide ligand (Crutchley & Naklicki, 1989). The asymmetric unit of the title compound is shown in Fig. 1. In the title compound, the Cd^II^ atom lies on an inversion center and has a distorted octahedral geometry (Fig. 2, Table 1). The coordination environment consists of four N atoms from 4-nitro-phenylcyanamide ligands in the equatorial plane and two O atoms from DMF molecules in the axial positions. The one-dimensional structure of the title compound is shown in Fig. 2.

### Experimental

4-Nitrophenylcynamide (Crutchley & Naklicki, 1989) (0.163 g, 1 mmol) was dissolved in methanol (25 ml) and was added slowly to a solution of cadmium(II) acetate (0.133 g, 0.5 mmol) in methanol (25 ml). The mixture was stirred for 3 hrs. The resulting solid was filtered off. Yellow needles of the title compound were obtained by n-hexane diffusion into a DMF solution of the title compound after 4 weeks.

### Refinement

H atoms were positioned geometrically and refined as riding atoms, with C—H = 0.93 (CH) and 0.96 (CH_3_) Å and with *U*_iso_(H) = 1.2(1.5 for methyl)*U*_eq_(C).

### Crystal data

**Table:**

[Cd(C_7_H_4_N_3_O_2_)_2_(C_3_H_7_NO)_2_]	*Z* = 1
*M**_r_* = 582.87	*F*(000) = 294
Triclinic, *P*1	*D*_x_ = 1.650 Mg m^−^^3^
Hall symbol: -P 1	Mo *K*α radiation, λ = 0.71073 Å
*a* = 5.6070 (11) Å	Cell parameters from 3150 reflections
*b* = 9.811 (2) Å	θ = 2.3–29.2°
*c* = 11.679 (2) Å	µ = 0.98 mm^−^^1^
α = 67.44 (3)°	*T* = 298 K
β = 81.93 (3)°	Needle, yellow
γ = 84.28 (3)°	0.45 × 0.10 × 0.08 mm
*V* = 586.7 (2) Å^3^	

### Data collection

**Table:**

Stoe IPDS 2T diffractometer	3150 independent reflections
Radiation source: fine-focus sealed tube	3038 reflections with *I* > 2σ(*I*)
Graphite monochromator	*R*_int_ = 0.066
ω scans	θ_max_ = 29.2°, θ_min_ = 2.3°
Absorption correction: numerical (*X-SHAPE* and *X-RED32*; Stoe & Cie, 2002)	*h* = −7→7
*T*_min_ = 0.887, *T*_max_ = 0.923	*k* = −13→13
6589 measured reflections	*l* = −15→16

### Refinement

**Table:**

Refinement on *F*^2^	Primary atom site location: structure-invariant direct methods
Least-squares matrix: full	Secondary atom site location: difference Fourier map
*R*[*F*^2^ > 2σ(*F*^2^)] = 0.044	Hydrogen site location: inferred from neighbouring sites
*wR*(*F*^2^) = 0.114	H-atom parameters constrained
*S* = 1.11	*w* = 1/[σ^2^(*F*_o_^2^) + (0.0672*P*)^2^ + 0.308*P*] where *P* = (*F*_o_^2^ + 2*F*_c_^2^)/3
3150 reflections	(Δ/σ)_max_ < 0.001
162 parameters	Δρ_max_ = 0.82 e Å^−^^3^
0 restraints	Δρ_min_ = −0.89 e Å^−^^3^

### Special details

**Table:**

Geometry. All e.s.d.'s (except the e.s.d. in the dihedral angle between two l.s. planes) are estimated using the full covariance matrix. The cell e.s.d.'s are taken into account individually in the estimation of e.s.d.'s in distances, angles and torsion angles; correlations between e.s.d.'s in cell parameters are only used when they are defined by crystal symmetry. An approximate (isotropic) treatment of cell e.s.d.'s is used for estimating e.s.d.'s involving l.s. planes.
Refinement. Refinement of *F*^2^ against ALL reflections. The weighted *R*-factor *wR* and goodness of fit *S* are based on *F*^2^, conventional *R*-factors *R* are based on *F*, with *F* set to zero for negative *F*^2^. The threshold expression of *F*^2^ > σ(*F*^2^) is used only for calculating *R*-factors(gt) *etc*. and is not relevant to the choice of reflections for refinement. *R*-factors based on *F*^2^ are statistically about twice as large as those based on *F*, and *R*- factors based on ALL data will be even larger.

### Fractional atomic coordinates and isotropic or equivalent isotropic displacement parameters (Å^2^)

**Table:**

	*x*	*y*	*z*	*U*_iso_*/*U*_eq_	
Cd1	0.0000	1.0000	0.0000	0.03092 (12)	
O1	−0.1591 (9)	0.2034 (5)	0.4718 (4)	0.0897 (14)	
O2	0.1354 (7)	0.1984 (4)	0.5719 (3)	0.0705 (10)	
O3	0.0654 (5)	1.1593 (3)	0.0984 (3)	0.0493 (6)	
N1	0.6524 (5)	0.9163 (3)	0.1262 (3)	0.0404 (6)	
N2	0.2627 (5)	0.8176 (3)	0.1293 (3)	0.0362 (5)	
N3	0.0180 (6)	0.2570 (4)	0.4842 (3)	0.0511 (8)	
N4	0.3441 (5)	1.1798 (4)	0.2129 (3)	0.0417 (6)	
C1	0.2102 (5)	0.6769 (3)	0.2158 (3)	0.0326 (6)	
C2	0.3446 (6)	0.6018 (4)	0.3172 (3)	0.0392 (7)	
H2	0.4765	0.6457	0.3264	0.047*	
C3	0.2838 (7)	0.4640 (4)	0.4033 (3)	0.0437 (7)	
H3	0.3748	0.4149	0.4694	0.052*	
C4	0.0864 (6)	0.4002 (4)	0.3899 (3)	0.0387 (7)	
C5	−0.0458 (6)	0.4682 (4)	0.2903 (4)	0.0446 (8)	
H5	−0.1761	0.4225	0.2818	0.053*	
C6	0.0172 (6)	0.6057 (4)	0.2026 (4)	0.0413 (7)	
H6	−0.0697	0.6511	0.1344	0.050*	
C7	0.4701 (5)	0.8656 (4)	0.1296 (3)	0.0338 (6)	
C8	0.1668 (6)	1.1147 (4)	0.1947 (4)	0.0412 (7)	
H8	0.1137	1.0288	0.2590	0.049*	
C9	0.4414 (9)	1.3125 (5)	0.1153 (5)	0.0584 (10)	
H9A	0.6050	1.2913	0.0873	0.088*	
H9B	0.4371	1.3892	0.1476	0.088*	
H9C	0.3461	1.3444	0.0466	0.088*	
C10	0.4716 (9)	1.1095 (6)	0.3235 (5)	0.0598 (11)	
H10A	0.3882	1.0246	0.3806	0.090*	
H10B	0.4778	1.1784	0.3631	0.090*	
H10C	0.6327	1.0793	0.2994	0.090*	

### Atomic displacement parameters (Å^2^)

**Table:**

	*U*^11^	*U*^22^	*U*^33^	*U*^12^	*U*^13^	*U*^23^
Cd1	0.01969 (14)	0.03075 (16)	0.03821 (18)	−0.00441 (9)	−0.00930 (10)	−0.00556 (12)
O1	0.092 (3)	0.066 (2)	0.089 (3)	−0.048 (2)	−0.027 (2)	0.011 (2)
O2	0.073 (2)	0.0528 (18)	0.0584 (19)	−0.0116 (16)	−0.0145 (16)	0.0133 (15)
O3	0.0510 (14)	0.0441 (14)	0.0569 (16)	−0.0031 (11)	−0.0226 (12)	−0.0175 (12)
N1	0.0274 (11)	0.0406 (14)	0.0439 (15)	−0.0065 (10)	−0.0072 (10)	−0.0035 (12)
N2	0.0253 (11)	0.0335 (13)	0.0425 (14)	−0.0060 (9)	−0.0082 (10)	−0.0037 (11)
N3	0.0499 (17)	0.0391 (16)	0.0529 (18)	−0.0092 (13)	−0.0037 (14)	−0.0038 (14)
N4	0.0381 (13)	0.0435 (15)	0.0451 (15)	−0.0046 (11)	−0.0100 (11)	−0.0159 (13)
C1	0.0256 (12)	0.0321 (14)	0.0362 (14)	−0.0014 (10)	−0.0073 (10)	−0.0072 (11)
C2	0.0347 (14)	0.0401 (16)	0.0398 (16)	−0.0078 (12)	−0.0126 (12)	−0.0075 (13)
C3	0.0431 (17)	0.0400 (17)	0.0389 (17)	−0.0069 (13)	−0.0139 (13)	−0.0003 (13)
C4	0.0380 (15)	0.0302 (14)	0.0436 (17)	−0.0045 (11)	−0.0024 (13)	−0.0092 (13)
C5	0.0341 (15)	0.0359 (16)	0.059 (2)	−0.0078 (12)	−0.0133 (14)	−0.0081 (15)
C6	0.0332 (14)	0.0350 (15)	0.0507 (19)	−0.0046 (11)	−0.0181 (13)	−0.0052 (14)
C7	0.0256 (12)	0.0349 (14)	0.0340 (14)	0.0007 (10)	−0.0073 (10)	−0.0045 (11)
C8	0.0410 (16)	0.0386 (16)	0.0467 (18)	−0.0055 (13)	−0.0073 (13)	−0.0172 (14)
C9	0.059 (2)	0.054 (2)	0.060 (2)	−0.0214 (19)	−0.0045 (19)	−0.015 (2)
C10	0.056 (2)	0.072 (3)	0.056 (2)	0.001 (2)	−0.0242 (19)	−0.024 (2)

### Geometric parameters (Å, º)

**Table:**

Cd1—N1^i^	2.287 (3)	C2—C3	1.381 (5)
Cd1—O3	2.347 (3)	C2—H2	0.9300
Cd1—N2	2.383 (3)	C3—C4	1.380 (5)
O1—N3	1.219 (5)	C3—H3	0.9300
O2—N3	1.214 (5)	C4—C5	1.377 (5)
O3—C8	1.238 (5)	C5—C6	1.388 (5)
N1—C7	1.170 (4)	C5—H5	0.9300
N1—Cd1^ii^	2.287 (3)	C6—H6	0.9300
N2—C7	1.299 (4)	C8—H8	0.9300
N2—C1	1.392 (4)	C9—H9A	0.9600
N3—C4	1.461 (4)	C9—H9B	0.9600
N4—C8	1.316 (4)	C9—H9C	0.9600
N4—C9	1.456 (5)	C10—H10A	0.9600
N4—C10	1.460 (5)	C10—H10B	0.9600
C1—C6	1.401 (4)	C10—H10C	0.9600
C1—C2	1.409 (4)		
			
N1^i^—Cd1—N1^iii^	180.000 (1)	C3—C2—H2	119.5
N1^i^—Cd1—O3	86.19 (12)	C1—C2—H2	119.5
N1^iii^—Cd1—O3	93.81 (12)	C4—C3—C2	119.2 (3)
N1^i^—Cd1—O3^iv^	93.81 (12)	C4—C3—H3	120.4
N1^iii^—Cd1—O3^iv^	86.19 (12)	C2—C3—H3	120.4
O3—Cd1—O3^iv^	180.00 (10)	C5—C4—C3	121.5 (3)
N1^i^—Cd1—N2^iv^	95.72 (10)	C5—C4—N3	119.8 (3)
N1^iii^—Cd1—N2^iv^	84.28 (10)	C3—C4—N3	118.7 (3)
O3—Cd1—N2^iv^	90.74 (10)	C4—C5—C6	119.4 (3)
O3^iv^—Cd1—N2^iv^	89.26 (10)	C4—C5—H5	120.3
N1^i^—Cd1—N2	84.28 (10)	C6—C5—H5	120.3
N1^iii^—Cd1—N2	95.72 (10)	C5—C6—C1	120.8 (3)
O3—Cd1—N2	89.26 (10)	C5—C6—H6	119.6
O3^iv^—Cd1—N2	90.74 (10)	C1—C6—H6	119.6
N2^iv^—Cd1—N2	180.0	N1—C7—N2	176.4 (3)
C8—O3—Cd1	121.5 (2)	O3—C8—N4	124.9 (4)
C7—N1—Cd1^ii^	142.5 (3)	O3—C8—H8	117.6
C7—N2—C1	116.9 (3)	N4—C8—H8	117.6
C7—N2—Cd1	113.9 (2)	N4—C9—H9A	109.5
C1—N2—Cd1	128.50 (19)	N4—C9—H9B	109.5
O2—N3—O1	123.1 (4)	H9A—C9—H9B	109.5
O2—N3—C4	119.1 (3)	N4—C9—H9C	109.5
O1—N3—C4	117.7 (4)	H9A—C9—H9C	109.5
C8—N4—C9	120.7 (3)	H9B—C9—H9C	109.5
C8—N4—C10	120.8 (4)	N4—C10—H10A	109.5
C9—N4—C10	117.9 (4)	N4—C10—H10B	109.5
N2—C1—C6	119.5 (3)	H10A—C10—H10B	109.5
N2—C1—C2	122.5 (3)	N4—C10—H10C	109.5
C6—C1—C2	118.0 (3)	H10A—C10—H10C	109.5
C3—C2—C1	121.1 (3)	H10B—C10—H10C	109.5
			
N1^i^—Cd1—O3—C8	−96.1 (3)	C6—C1—C2—C3	−1.9 (5)
N1^iii^—Cd1—O3—C8	83.9 (3)	C1—C2—C3—C4	−0.7 (6)
N2^iv^—Cd1—O3—C8	168.2 (3)	C2—C3—C4—C5	2.5 (6)
N2—Cd1—O3—C8	−11.8 (3)	C2—C3—C4—N3	−177.6 (4)
N1^i^—Cd1—N2—C7	35.1 (3)	O2—N3—C4—C5	−179.7 (4)
N1^iii^—Cd1—N2—C7	−144.9 (3)	O1—N3—C4—C5	−1.2 (6)
O3—Cd1—N2—C7	−51.2 (3)	O2—N3—C4—C3	0.4 (6)
O3^iv^—Cd1—N2—C7	128.8 (3)	O1—N3—C4—C3	178.9 (5)
N1^i^—Cd1—N2—C1	−155.1 (3)	C3—C4—C5—C6	−1.5 (6)
N1^iii^—Cd1—N2—C1	24.9 (3)	N3—C4—C5—C6	178.6 (4)
O3—Cd1—N2—C1	118.6 (3)	C4—C5—C6—C1	−1.2 (6)
O3^iv^—Cd1—N2—C1	−61.4 (3)	N2—C1—C6—C5	−177.2 (3)
C7—N2—C1—C6	−165.8 (3)	C2—C1—C6—C5	2.9 (5)
Cd1—N2—C1—C6	24.6 (5)	Cd1—O3—C8—N4	131.9 (3)
C7—N2—C1—C2	14.0 (5)	C9—N4—C8—O3	−1.7 (6)
Cd1—N2—C1—C2	−155.5 (3)	C10—N4—C8—O3	−172.6 (4)
N2—C1—C2—C3	178.2 (3)		

Symmetry codes: (i) −*x*+1, −*y*+2, −*z*; (ii) *x*+1, *y*, *z*; (iii) *x*−1, *y*, *z*; (iv) −*x*, −*y*+2, −*z*.
